# Regional inequality in the Janani Suraksha Yojana coverage in India: a geo-spatial analysis

**DOI:** 10.1186/s12939-020-01366-2

**Published:** 2021-01-07

**Authors:** Prem Shankar Mishra, Pradeep Kumar, Shobhit Srivastava

**Affiliations:** 1 Institute for Social and Economic Change, Bengaluru, Karnataka 560072 India; 2 International Institute for Population Sciences, Mumbai, Maharashtra 400088 India

**Keywords:** Janani Suraksha Yojana, Spatial analysis, LISA, India

## Abstract

**Introduction:**

Although India has made significant progress in institutional delivery after the implementation of the National Rural Health Mission under which the Janani Suraksha Yojana (JSY) is a sub-programme which played a vital role in the increase of institutional delivery in public facilities. Therefore, this paper aims to provide an understanding of the JSY coverage at the district level in India. Further, it tries to carve out the factors responsible for the regional disparity of JSY coverage at district levels.

**Methods:**

The study used the National Family Health Survey data, which is a cross-sectional survey conducted in 2015–16, India. The sample size of this study was 148,145 women aged 15–49 years who gave last birth in the institution during 5 years preceding the survey. Bivariate and multivariate regression analysis was used to fulfill the study objectives. Additionally, Moran’s I statistics and bivariate Local Indicator for Spatial Association (LISA) maps were used to understand spatial dependence and clustering of JSY coverage. Ordinary least square, spatial lag and spatial error models were used to examine the correlates of JSY utilization.

**Results:**

The value of spatial-autocorrelation for JSY was 0.71 which depicts the high dependence of the JSY coverage over districts of India. The overall coverage of JSY in India is 36.4% and it highly varied across different regions, districts, and even socioeconomic groups. The spatial error model depicts that if in a district the women with no schooling status increase by 10% then the benefits of JSY get increased by 2.3%. Similarly, if in a district the women from poor wealth quintile, it increases by 10% the benefits of JSY also increased by 4.6%. However, the coverage of JSY made greater imperative to understand it due to its clustering among districts of specific states only.

**Conclusion:**

It is well reflected in the EAGs states in terms of spatial-inequality in service coverage. There is a need to universalize the JSY programme at a very individual level. And, it is required to revisit the policy strategy and the implementation plans at regional or district levels.

## Introduction

Despite given efforts by international, national, and local governments and agencies, the utilization status of maternal and child health (MCH) care services is still low in many developing countries, including India [1–6]. Although India has made considerable progress in reducing maternal mortality through the interventions of different health policies and programs, the national rural health mission (NRHM) is one of them and act as mandating multi-strategic programme interventions to promote health care accessibility while reducing health inequity across the groups [7, 8], however, kinds of literature show that the effectiveness and efficiency of the programs are not the same across socio-economic groups and regions that led to slow, uneven and unequal distribution of health and health care practices [9–13]. Further, a large proportion of women and children in low-and middle-income countries are still, not covered under the essential health care services [13–18] and particularly those who are from the poor and marginalized groups of the communities [10, 19, 20]. India is also facing the same issue of health inequality, and even worse in the case of MCH care services [8, 20, 21].

Furthermore, in India, huge health disparities exist across different socio-economic groups, regions, states, and districts level among women and children. And it is due to low accessing and under-utilizing of maternal and child health care services [22]. States like Uttar Pradesh, Bihar, Madhya Pradesh, Rajasthan, Jharkhand, Chhattisgarh, Uttarakhand, and Odisha are going through tremendous inequality in accessing equitable health care services [11, 22, 23]. These states are also together named as EAGs (Empowered Action Group) states, with low performing in socio-economic and health indicators, and that eventually lead to high maternal and child mortality compared to other states [17, 22–27].

Further, higher maternal mortality rates and its variations across socio-geographical regions show that there is inaccessibility, unavailability, and unaffordability of essential maternity services that lead to under-utilization of MCH services among the poor and marginalized women [17, 19]. For example, institutional delivery is an important maternity care service that prevents maternal and neonatal mortality. In India, still, 21% of childbirth delivery occurs at home [23]. Although, institutional delivery in India has increased to 79% in 2015 from 39% in 2005; however, still, the gap has remained wide and persistent across socio-economic groups, regions, and states [23]. For example, women belong to a higher wealth quintile have gone to 95% of institutional delivery as compared to 60% of a lower wealth quintile mothers. Similar differences can also be found in mother’s education levels [23]. Therefore, for plummeting health inequity and fostering health equality development by promoting institutional delivery, the *Janani Suraksha Yojana* (JSY) or ‘Safe Motherhood scheme’ was introduced in 2005, under the National Rural Health Mission (NRHM), in which the poor and marginalized women are provided with an incentive for delivering their child in public health facilities. It is a conditional cash transfer scheme to promote institutional delivery in order to reduce mother and child deaths [22, 28].

The economic burden is one of the most important factors that restrict poor pregnant women from delivering their childbirth at health institutions [28–32]. Further, socio-economically vulnerable and marginalized women also suffer from multiple deprivations/vulnerabilities in seeking maternal health care services, such as prenatal, natal, post-natal care, and child immunization [25–27, 32–34]. The JSY scheme is one of the most far-reaching demand-side financing programs in the world [12, 21, 35]. And, it is associated with increasing institutional delivery among the most deprived groups of people. Further, it has significantly improved institutional delivery in the low performing states (EAGs states) in the last one decade 2005–2015, however, the state and regional level variations still continue to persist [20, 21, 23, 24, 35–38].

The provision of conditioning JSY cash payment scheme to all pregnant women is marked as an irregularity to the beneficiaries and it has been found that after a decade of implementation of the JSY scheme, a huge gap persists in terms of coverage and utilization [5, 7, 38], not only across socio-economic groups but also at regional and district-levels [7, 20, 35]. Several studies found that the increasing trend of inequity and inequality in access to JSY services and its coverage has created policy concerns [7, 8, 20, 29]; therefore, it requires putting forth many questions against the policies and programme for its overall coverage [4, 7]. Further, there is also supply-side barriers women face in accessing JSY services [6]. Women belong to a marginalized and disadvantaged community are unable to meet the required MCH services available in the public domains in India [29, 32, 34, 39], although the community health workers (CHWs) are the key to improve the service coverage in the community, however, there are evidences show that CHWs are biased in providing healthcare services in the community across the social groups [27, 39, 40]. The literature also shows that there is a significant variation in coverage of health policy and programs interventions across the communities due to unawareness and lack of knowledge [5, 20, 34, 41]. Due to the policy coverage gap and lack of programme effectiveness across the groups, regions, and states have made substantial increments in the health disparity. It is evident in the study conducted by Vellakkal et al., that the use of JSY conditioning cash transfer during pregnancy is varied considerably across socio-economic groups, and not all eligible women get access to it [34]. Moreover, it also varies across geographic regions and states in India [6, 20, 22]. Spatial disparity matters in the MCH services coverage and its utilization [23, 42–45]. Studies show that there is a strong correlation between the proximate determinants of spatial clustering and service coverage [43–45].

As it was found that a huge gap persists in the JSY coverage across various socio-economic groups, states, and regions of India. Therefore, this paper aims to provide an understanding of the JSY coverage at the district level. Moreover, it tries to carve out the factors responsible for regional disparity for JSY coverage at the district level. The study hypothesized that there was no spatial auto-correlation between JSY coverage and districts of India.

### Janani Suraksha Yojana (Safe Motherhood Programme)

India has launched several health policies and programs to protect mother and child health and to improve their survival. India’s flagship scheme of JSY launched in 2005 under the auspicious program of the National Rural Health Mission (NRHM) with a particular focus on reducing maternal and infant mortality through promoting antenatal, natal, and postnatal care. JSY is a safe motherhood intervention under the NRHM being implemented with the objective to reduce maternal and neonatal mortality by promoting institutional delivery among poor pregnant women. It is a 100% centrally sponsored scheme and it integrates cash assistance with delivery and post-delivery care [22]. It is a conditioning cash-incentive scheme that promotes pregnant women to deliver their children at public health institutions. Since, in India, one-fifth of childbirths still take place at home delivery [23]. It ensures safe delivery to all women who belong to Scheduled Castes, Scheduled Tribes, and those women who are living below the poverty line (BPL) with the age of 19 years and above during delivery. The ASHA as a community health worker (Accredited Social Health Activist) acts as an intermediate person to track from pregnancy to childbirth and postnatal care in the community in this scheme. In this way, ASHA is engaged with the JSY scheme to set up a linkage between the government health system and the beneficiary woman [22]. Each beneficiary registered under this scheme must have a JSY card along with an MCH card.

## Methods

The data from the National Family Health Survey round four (NFHS-4) was used to understand the spatial pattern and correlates affecting the JSY service utilization in India. NFHS is a cross-sectional national representative survey, conducted in 2015–16 under the stewardship of the Ministry of Health and Family Welfare (MoHFW), Government of India. The survey provides detailed information on population, fertility, family planning, reproductive right and health issue, HIV/AIDS, gender issues, women empowerment, and domestic violence. NFHS used a two-stage stratified sampling design in both rural and urban areas to give the estimates at state [46] as well as district level (640). In rural areas, villages were selected in the first stage using a Probability Proportional to Size (PPS) scheme. In the second stage, 22 HHs were selected using systematic sampling. In urban areas, census enumeration blocks (CEBs) were selected in the first stage using the PPS scheme, and in the second stage, 22 HHs were selected using systematic sampling. The detailed methodology and complete information on the survey design and data collection published elsewhere [23]. The survey collected information from 601,509 households, 699,686 women, and 112,122 men for the response rate of 98%, 97%, and 92% respectively. The study restricts sample size (*n*=148,145) to the women aged 15–49 years who gave last birth in the institution during 5 years preceding the survey.

### Outcome variable

The outcome variable for the analysis is the coverage (percentage) of the JSY scheme. The question was asked to the women ‘did you receive any financial assistance for delivery care? Further, the question was asked ‘from where did you get assistance? The responses were (a). Janani Suraksha Yojana (JSY), (b). Other Government Schemes, (c). Other. For the analysis purpose, the study made a dichotomous variable and which was coded as 1 ‘Yes (received JSY assistance)’ and 0 ‘No (did not received JSY assistance)’.

### Independent variable

The predictor variables for this study were women’s age, meeting with community health worker (CHW), education of the women, the wealth of the households, caste, religion, mass media exposure and place of residence. Age of the women was divided into two categories: less than 25 years and 25 years or more. Meeting with community health worker (CHW) was coded as ‘yes’ and ‘no’. Women’s educational level was categorized as no education and educated. A household wealth index was calculated in the survey by combining household amenities, assets and durables and characterizing households in a range varying from the poorest to the richest, corresponding to wealth quintiles ranging from the lowest to the highest. Further, the study grouped wealth of the household into two categories such as poor (included poorest and poorer) and non-poor (included middle, richer, and richest). Place of residence was given as rural and urban in the survey. Caste was divided into two categories: Scheduled Caste/Scheduled Tribe and other (included other backward class caste group). Religion was categorized as Hindu and non-Hindu (including Christian, Sikh, Buddhist/Neo-Buddhist, Jain, Jewish, Parsi/Zoroastrian, no religion, and other). Women’s exposure to mass media: how often they read newspapers, listened to the radio and watched television; responses on the frequencies were: almost every day, at least once a week, less than once a week, or not at all; women were considered to have any exposure to mass media if they had exposure to any of these sources and as having no exposure if they responded with ‘not at all’ for all three sources of media [44, 47].

### Statistical analysis

Bivariate and multivariate logistic regression analysis was used to analyze the data. Additionally, for spatial analysis in terms of coverage of JSY among women in India univariate and bivariate Moran’s I index measurements were used along with the usage of spatial regression models [48]. Spatial auto-correlation is being measured by using Moran’s I statistics. Spatial autocorrelation represents the extent to which data points are similar or dissimilar to their spatial neighbours [49–51].

Univariate Moran’s I measure the spatial auto-correlation of neighborhood values around a specific spatial location. It determines the extent of spatial non-stationary and clustering present in the data. Bivariate Moran’s I examine the local correlation between an outcome variable and certain characteristics of the region. While both univariate and bivariate Moran’s I aim to measure similarities and dissimilarities of spatial data, they are found to be less useful in case of uneven spatial clustering [48, 51]. The formula to calculate the Moran’s *I* statistic is as follows:

$$ \mathrm{Univariate}\ \mathrm{Moran}'\mathrm{s}\ \mathrm{I}=\frac{n}{S_O}\times \frac{\Sigma_i{\Sigma}_j{W}_{ij}\left({x}_i-\overline{X}\right)\left[{x}_j-\overline{X}\right]}{\Sigma_i{\left[{x}_i-\overline{X}\right]}^2} $$

Where x is the variable of interest and $\overline{X}$ is the mean of x; n is the number of spatial units; *W*_*ij*_ is the standardized weight matrix between observation i and j with zeroes on the diagonal; and *S*_*O*_ is the aggregate of all spatial weights, i.e. *S*_*O*_ = Σ_*i*_Σ_*j*_*W*_*ij*_

$$ \mathrm{Bivariate}\ \mathrm{Moran}'\mathrm{s}\ \mathrm{I}=\frac{n}{S_O}\times \frac{\Sigma_i{\Sigma}_j{W}_{ij}\left({x}_i-\overline{X}\right)\left[{Y}_j-\overline{Y}\right]}{\Sigma_i{\left[{y}_i-\overline{Y}\right]}^2} $$

Where x and y are the variables of interest; $\overline{X}$ is the mean of x; $\overline{Y}$ is the mean of y; n is the number of spatial units; *W*_*ij*_ is the standardized weight matrix between observation i and j with zeroes on the diagonal; and *S*_*O*_ is the aggregate of all spatial weights, i.e. *S*_*O*_ = Σ_*i*_Σ_*j*_*W*_*ij*_.

Value of Moran’s- I ranges from − 1 (indicating perfect dispersion) to + 1 (perfect correlation). A zero value indicates a random spatial pattern. Negative (positive) values indicate a negative (positive) spatial autocorrelation. Positive autocorrelation indicates that points with similar attribute values are closely distributed in space, whereas negative spatial autocorrelation indicates that closely associated points are more dissimilar [45, 48, 50, 51].

Univariate LISA calculates the spatial-correlation of neighborhood values around the specific spatial location [51]. It determines the extent of spatial randomness and clustering present in the data. The measure [*I*_*i*_] is given by the following:

$$ \mathrm{Univariate}\ \mathrm{LISA}:{I}_i=\frac{n.\left[{x}_i-\overline{X}\right]}{\Sigma_i{\left[{x}_i-\overline{X}\right]}^2}{\Sigma}_j{w}_{ij}\left[{x}_j-\overline{X}\right] $$

Bivariate Local Indicator of Spatial Association (LISA) measures were estimated to analyze the association of certain characteristics of regions with JSY coverage. The bivariate LISA presented as below:

$$ \mathrm{Bivariate}\ \mathrm{LISA}:{I}_i=\frac{n.\left[{x}_i-\overline{X}\right]}{\Sigma_i{\left[{y}_i-\overline{Y}\right]}^2}{\Sigma}_j{w}_{ij}\left[{y}_i-\overline{Y}\right] $$

Four types of spatial auto-correlation were generated based on the four quadrants of Moran’s I scatter plots which are defined as follows:

- Hot Spots: districts with high values, with similar neighbors (High-High).
- Cold Spots: districts with low values, with similar neighbors (Low-Low).
- Spatial Outliers: districts with high values, but with low-value neighbors (High-Low) and districts with low values, but with higher values of neighbors (Low-High).

The spatial weights W_ij_ are non-zero when i and j are neighbors, else it remains zero [49, 50]. The weight used in the analysis is Queen Contiguity weights which represents whether spatial units share the boundary or not. If the set of boundary points of unit I is denoted by the band (i), then the Queen Contiguity Weight is defined by:

$$ \mathrm{Wij}=\left\{\begin{array}{c}1, bnd(i)\cap bnd\ (j)\ne \varnothing \\ {}0, bnd(i)\cap bnd\ (j)\ne \varnothing \end{array}\right. $$

However, this allows the possibility that spatial units share only a single boundary point (such as a shared corner point on a grid of spatial units). Hence a stronger condition is to require that some *positive* portion of their boundary be shared.

In order to determine the significant correlates of coverage of *Janani Suraksha Yojana* (JSY) in India, a set of regression models had been used. The spatial ordinary least square (OLS) regression model was used to see the extent of autocorrelation in the error term. Since the OLS confirmed spatial autocorrelation in its error term for the dependent variable, we further estimated the spatial lag model (SLM) and spatial error model (SEM) [48, 50]***.*** The underlying assumption of a spatial lag model is that the observations of the outcome variable are affected in the neighborhood areas whereas the spatial error model is used to consider the effect of those variables which are absent in the regression model but had an effect on the outcome variable. The basic difference between the two models is that the spatial lag model unlike the spatial error model does not consider the spatial dependence of the error term.

The basic equation for OLS is as follows:

$$ \mathrm{Y}=\upalpha +\mathrm{BX}+\upvarepsilon $$

Where Y is the outcome variable, X is the vector of predictor variables and α is the model intercept and β is the corresponding coefficient vector.

The spatial lag model suggests that the units are spatially dependent on each other and lagging to each in the nearby spatial locations [48, 51]. A typical spatial lag model can be written as follows:

$$ {Y}_i=\delta \sum \limits_{j\ne 1}{W}_{ij}{Y}_j+\beta {X}_j+{\varepsilon}_j $$

Here *Y*_*i*_ denotes the JSY coverage for the *i*^*th*^ district, δ is the spatial autoregressive coefficient, *W*_*ij*_ denotes the spatial weight of proximity between district i and j, *Y*_*j*_ is the JSY coverage in the *j*^*th*^ district, *β*_*j*_ denotes the coefficient, *X*_*j*_ is the predictor variable and εj is the residual.

The spatial error model, on the other hand, considers the contribution of omitted variables that are not included in the model but can have a significant effect in the analysis [51]***.*** A Spatial Error Model (SEM) is expressed as follows:

$$ {Y}_i=\beta {X}_j+\lambda \sum \limits_{j\ne 1}{\mathrm{W}}_{ij}{Y}_j{\varepsilon}_j+{\varepsilon}_i $$

Here, *Y*_*i*_ denotes the JSY coverage for the *i*^*th*^ district, λ is the spatial autoregressive coefficient, *W*_*ij*_ denotes the spatial weight of proximity between district i and j, *Y*_*j*_ is JSY coverage in the *j*^*th*^ district, *β*_*j*_ denotes the coefficient, *X*_*j*_ is the predictor variable and *ε*_*i*_ is the residual.

## Results

### Background analysis

Table 1 represents the socio-economic profile of the study population in India. As per 2015–16 estimates, 36.4% of women in India got benefited from *Janani Suraksha Yojana* (JSY). About 68.9% of women were aged 25 years and more. Nearly, 51.9% of women met community health worker (CHW). Of the total women selected, 21.6% were having no schooling. Every 3 in 10 women were from the Scheduled Caste/Scheduled Tribe caste category. About 8 in 10 women were from the Hindu religion. About 37.6% of women belonged to the poor wealth quintile and 19% of women had no media exposure. Further, about 67% of women belonged to rural areas in India.

**Table 1:** Socio-demographic profile of the study population in India, 2015–16

Variables	*N*=148,185
	(n(weighted %))
**Received Janani Suraksha Yojana**	
No	84,520 (63.6)
Yes	63,665 (36.4)
**Age (in years)**	
Less than 25	50,485 (36.1)
25 or more	97,700 (63.9)
**Met with CHW**	
No	70,723 (48.1)
Yes	77,462 (51.9)
**Educational level**	
No schooling	33,814 (21.6)
Educated	114,371 (78.4)
**Caste**	
Scheduled Caste/Scheduled Tribe	51,896 (31.0)
Non-Scheduled Caste/Scheduled Tribe	90,060 (69.0)
**Religion**	
Hindu	111,810 (80.6)
Non-Hindu	36,375 (19.4)
**Wealth quintile**	
Poor	59,298 (37.6)
Non-poor	88,887 (62.4)
**Mass media exposure**	
No exposure	29,725 (19.0)
Some exposure	118,460 (81.0)
**Place of residence**	
Urban	42,215 (33.1)
Rural	105,970 (66.9)

*%* percentage, *N* Sample, *CHW* Community health worker

Table 2 depicts bivariate and logistic regression analysis to find an association between JSY and background factors in India, 2015–16. Women aged 25 years and more were 6% significantly more likely to receive the benefit of JSY (OR: 1.06; *p*< 0.01) than women aged 24 years or less. Women who met CHW were 71% significantly more likely to receive the benefit of JSY (OR: 1.71; *p*< 0.01) than women who did not met CHW. Women who were educated, they were having significantly lower odds for receiving JSY benefits in reference to women who had no schooling (OR: 0.80, *p*< 0.01). Odds for JSY benefits were higher among women from the Scheduled Caste/Scheduled Tribe category than women from non-Scheduled Caste/ Scheduled Tribe (OR: 0.80, *p*< 0.01). Women from the non-Hindu religion were 25% significantly less likely to receive benefits from JSY in comparison to women from the Hindu religion (OR: 0.75, *p*< 0.01). Women from non-poor wealth quintiles were 52% significantly less likely to receive JSY benefits in comparison to women from the poor wealth quintile (OR: 0.48, *p*< 0.01). Women who had some media exposure had lower odds of receiving JSY benefits than women who had no media exposure (OR: 0.90, *p*< 0.01). Odds for receiving JSY benefits were higher for women from a rural place of residence than women from urban areas (OR:1.56, *p*< 0.01).

**Table 2:** Results from bivariate and logistic regression analysis for JSY utilization by background factors in India, 2015–16

Variables	JSY (%)	OR (95% C.I.)
**Age (in years)**		
Less than 25	36.6	Ref.
25 or more	36.3	1.06***(1.03–1.08)
**Met with CHW**		
No	29.7	Ref.
Yes	42.6	1.71***(1.67–1.75)
**Educational level**		
No schooling	51.4	Ref.
Educated	32.3	0.78***(0.76–0.81)
**Caste**		
Scheduled Caste/Scheduled Tribe	44.3	Ref.
Non-Scheduled Caste/Scheduled Tribe	33.0	0.82***(0.80–0.84)
**Religion**		
Hindu	37.9	Ref.
Non-Hindu	29.9	0.76***(0.74–0.79)
**Wealth quintile**		
Poor	53.2	Ref.
Non-poor	26.2	0.48***(0.47–0.49)
**Mass media exposure**		
No exposure	54.4	Ref.
Some exposure	32.12	0.85***(0.83–0.88)
**Place of residence**		
Urban	21.4	Ref.
Rural	43.8	1.46***(1.42–1.50)

*JSY:* Janani Suraksha Yojana, *OR:* Odds Ratio, *CI:* Confidence Interval, ***if *p*< 0.01, *Ref:* Reference category, *%:* percentage, *CHW:* Community health worker

Table 3 presents the values of univariate and bivariate Moran’s I statistics. Univariate Moran’s I statistics represent the spatial auto-correlation of outcome and predictor variables. The value of spatial-autocorrelation for JSY was 0.71 which depicts high dependence of the outcome variable over districts of India. Additionally, the highest Moran’s I value among predictor variables was witnessed by women from the poor wealth quintile (0.75) followed by women from the Hindu religion (0.74) and women who had no mass media exposure (0.72). It was found that the spatial auto-correlation of JSY and women with no schooling was 0.35 and that with women from poor wealth quintile was 0.52. Additionally, the spatial auto-correlation of JSY and women from rural areas was 0.31, and women who had no media exposure were 0.42.

**Table 3:** Univariate and Bivariate Moron’s I Values for outcome and predictors in India, 2015–16

Variables	Univariate	Bivariate
		Janani Suraksha Yojana
Janani Suraksha Yojana (%)	0.71 (0.001)	–
Age (Less than 25 years) (%)	0.61 (0.001)	0.07 (0.001)
Met with CHW (%)	0.55 (0.001)	0.13 (0.001)
No schooling (%)	0.71 (0.001)	0.35 (0.001)
Poor wealth quintile (%)	0.75 (0.001)	0.52 (0.001)
Rural place of residence (%)	0.41 (0.001)	0.31 (0.001)
Scheduled Caste/Scheduled Tribe (%)	0.60 (0.001)	0.06 (0.001)
Hindu (%)	0.74 (0.001)	0.11 (0.001)
No mass media exposure (%)	0.72 (0.001)	0.42 (0.001)

*%:* Percentage, *CHW:* Community Health Worker

Table 4 provides estimates for spatial regression estimates for JSY and its predictors for 640 districts of India. From the OLS estimates, it was confirmed that women aged less than 25 years (β: − 0.614, *p*< 0.05), met CHW (β: 0.341, *p*< 0.05), no schooling status (β: 0.206, *p*< 0.05), poor wealth quintile (β: 0.637, *p*< 0.05) and rural place of residence (β: 0.035, *p*< 0.05) were found to be significant spatial predictors of JSY in India. The value of adjusted R-square was 0.54 and the value for AIC was found to be 5394.

**Table 4:** Spatial regression model for estimating spatial association between Janani Suraksha Yojana and background factors in India, 2015–16

Variables	OLS (***p***-value)	SLM (***p***-value)	SEM (***p***-value)
Age (Less than 25 years) (%)	−0.614 (0.000)	−0.317 (0.000)	−0.293 (0.000)
Met with CHW (%)	0.341 (0.000)	0.248 (0.000)	0.290 (0.000)
No schooling (%)	0.206 (0.004)	0.156 (0.000)	0.216 (0.000)
Poor wealth quintile (%)	0.637 (0.000)	0.245 (0.000)	0.439 (0.000)
Rural place of residence (%)	0.035 (0.350)	0.034 (0.165)	−0.017 (0.601)
Scheduled Caste/Scheduled Tribe (%)	0.008 (0.809)	0.024 (0.306)	0.028 (0.367)
Hindu (%)	0.044 (0.134)	0.027 (0.187)	0.069 (0.032)
No mass media exposure (%)	−0.094 (0.274)	−0.025 (0.670)	− 0.037 (0.607)
**N(Sample)**	640	640	640
**Rho**		0.67 (0.000)	
**Lambda**			0.80 (0.000)
**AIC**	5394.5	5004.2	4996.4
**Adjusted R**	0.54	0.78	0.79

*AIC:* Akaike information criterion, *OLS:* Ordinary least square, *SLM:* Spatial lag model, *SEM:* Spatial error model

The value of the lag coefficient was 0.67 (*p*< 0.01) from the SLM which signifies that a change in the JSY coverage in a particular district may statistically lag the rate of JSY coverage by 67% in the neighboring districts. In the spatial lag model, it was found that women aged 15–24 years (β:-0.317, *p*< 0.05), met CHW (β: 0.248, *p*< 0.05), no schooling status (β: 0.56, *p*< 0.05) and poor wealth quintile (β: 0.245, *p*< 0.05) were significantly associated with JSY coverage in India. The respective model splits the value of adjusted R-square as 0.78 and the value for AIC was found as 5004.

However, as per the theory of spatial regression models, the model with the lowest AIC value and highest R-square value is considered to be the best fit model. Therefore, as per our model estimates the lowest AIC and highest adjusted R-square value was found to be of spatial error model (SEM) which makes it the best fit model among all the three models. The spatial error model was having an AIC value of 4996 and an adjusted R-square value of 0.79. Interestingly the value of Lambda (spatial autoregressive coefficient)/error lag value was 0.80 (*p*< 0.01) which signifies that spatial influence on JSY coverage through the omitted variables not present in the SEM.

The model depicts that if in a district the women aged less than 25 years increases by 10% then benefit of JSY get significantly declined by about 2.9%. Similarly, in a particular district those women who met CHW get significantly increased by 10% then the benefit of JSY gets significantly increased by almost 2.9%. If in a district the women with no schooling status increases by 10% then the benefits of JSY get significantly increased by 2.2%. Similarly, if in a district the women from the poor wealth quintile increase by 10% the benefits of JSY also significantly increased by 4.4%. However, if in a district there is a 10% increase of women who had no mass media exposure then the JSY benefits get declined by 0.4%. Moreover, rural place of residence (β: 0.017, *p*> 0.05), Scheduled caste/Scheduled tribe status residence (β: 0.028, *p*> 0.05) and Hindu religion status residence (β: 0.069, *p*< 0.05) were positively associated with JSY estimates, but the results were not significant except for Hindu religion status. The results simply imply that districts with a higher percentage of women having no schooling status and belong to the poor wealth quintile had higher chances to get benefited from the JSY programme.

**Fig. 1:**
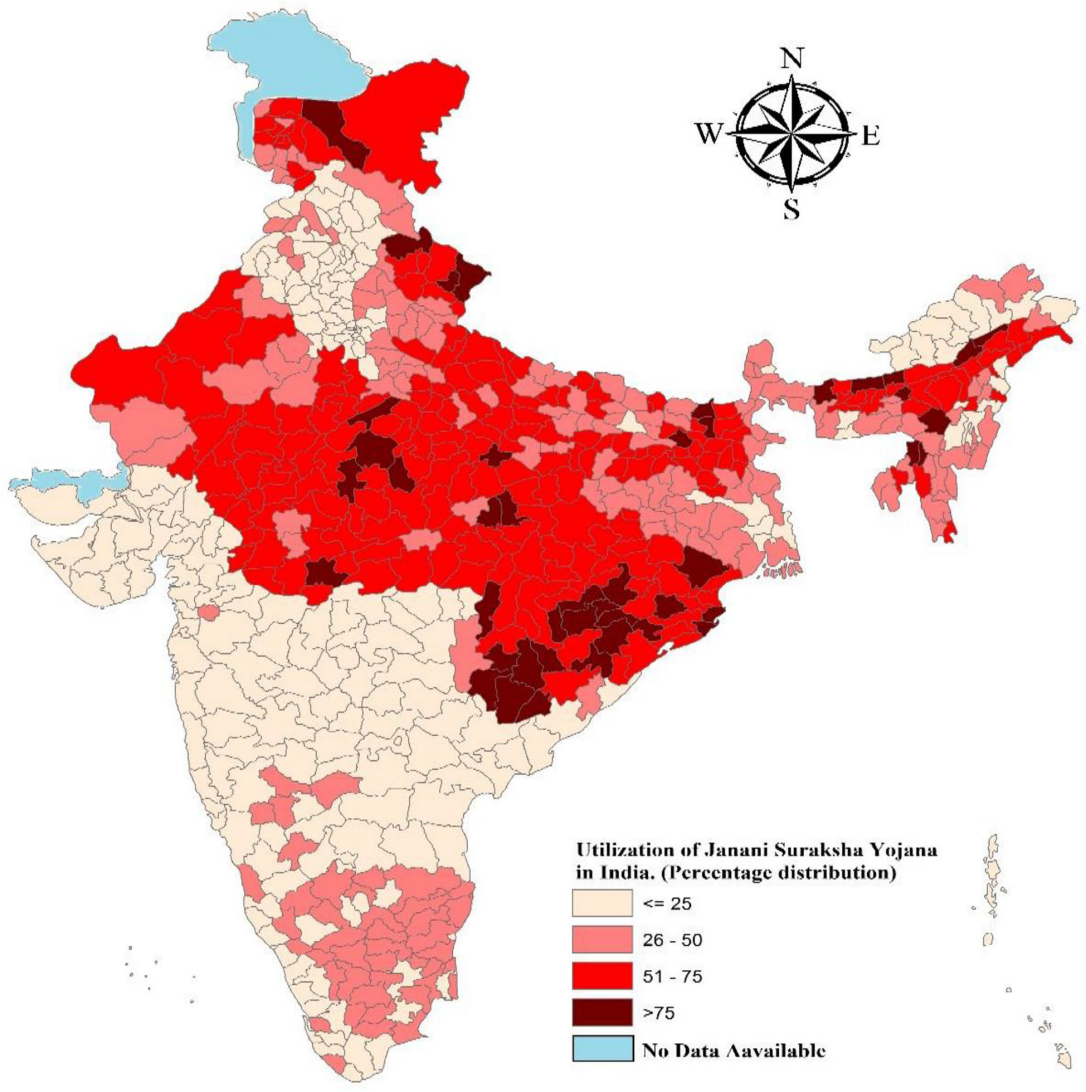
Percentage distribution of Janani Suraksha Yojana coverage among women in India

Figure 1 shows the coverage and spatial distribution of the JSY scheme across the districts of India. The colour pattern shows the spatial differences in the service utilization of the JSY scheme. Moreover, deeper colour indicates a higher proportion of JSY coverage and light colour indicates lower coverage. More than 50% of the women utilizing JSY services in the districts of Odisha, Chhattisgarh, Madhya Pradesh, Uttar Pradesh, Uttarakhand, Assam, and few districts of Rajasthan, Bihar, Jharkhand, and Meghalaya.

**Fig. 2:**
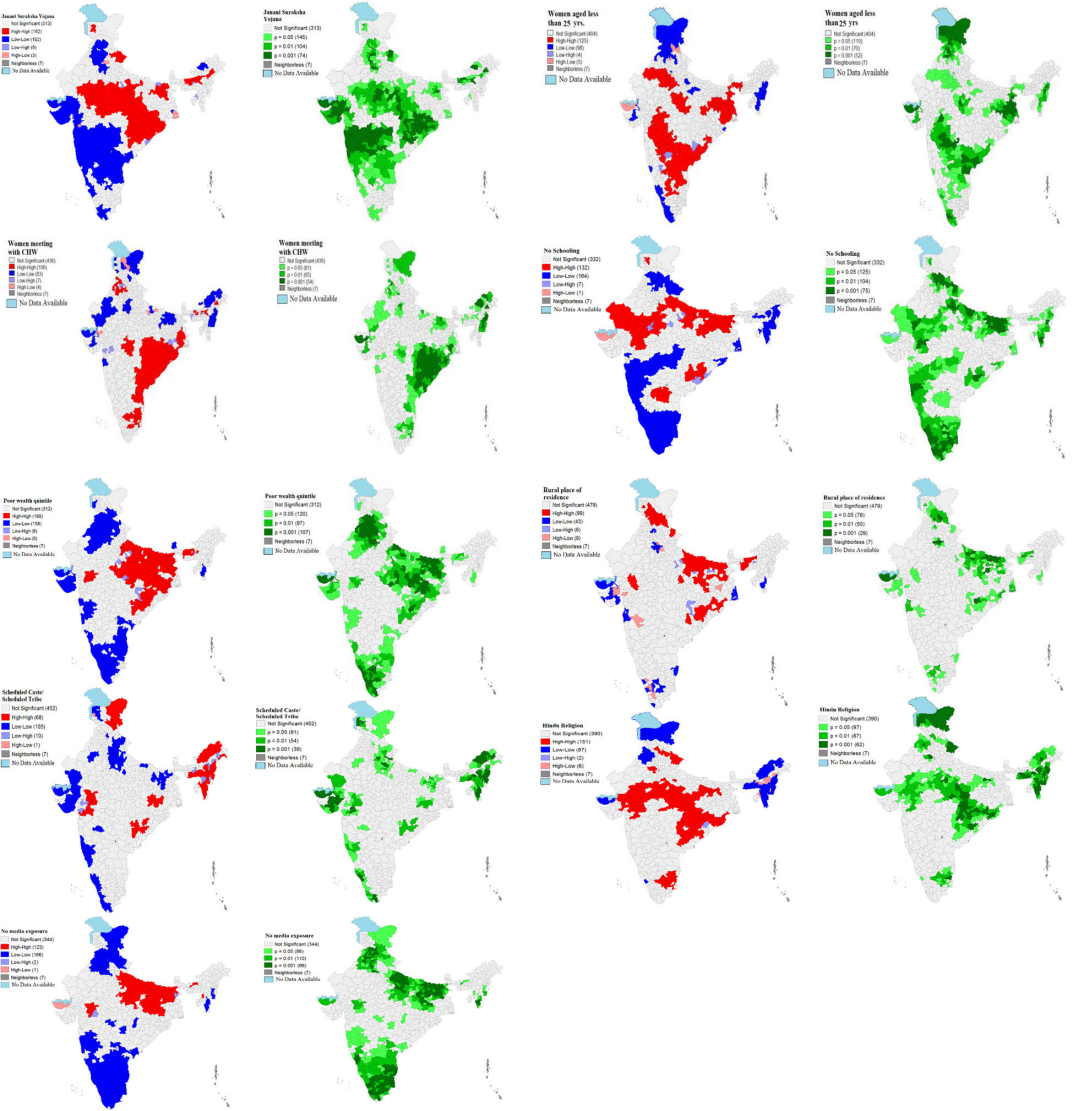
Univariate Local Indicator of Spatial Association (LISA) (cluster and significance) maps for dependent and outcome variables for districts of India, 2015–16. *CHW: Community health worker; Yrs.: Years*

Figure 2 represents univariate LISA (cluster and significance) maps for outcome and independent variables for districts of India, 2015–16. A significant high-high clustering of JSY service utilizing found in 162 districts, which belonged to the above-mentioned states. There were 162 cold spots in Gujarat, Maharashtra, Telangana Andhra Pradesh, Punjab, Chandigarh, and Haryana showed lower service utilization of the JSY scheme.

The high-high clustering (125 districts) for women aged less than 25 years was found in West Bengal, Jharkhand, Chhattisgarh, Rajasthan, Madhya Pradesh, Maharashtra, Andhra Pradesh, Odisha and Karnataka. The high-high clustering (106 districts) for women who met CHW was found in West-Bengal, Odisha, Madhya Pradesh, Madhya Pradesh, Andhra Pradesh, Karnataka and Tamil Nadu. On the other hand, no schooling hotspots (132 districts) were found in Rajasthan, Bihar, and few districts of Madhya Pradesh, Uttar Pradesh, and Odisha. While the clustering of poor women were more in the districts (160 districts) of Uttar Pradesh, Bihar, Jharkhand, Chhattisgarh, Odisha, and few parts of Assam. Furthermore, the hotspots (151 districts out of 640) of the Hindu religion were found in empowering action group states and the clustering of no mass media was high (123 districts) Uttar Pradesh, Bihar, Jharkhand, and few districts of Maharashtra.

**Fig. 3:**
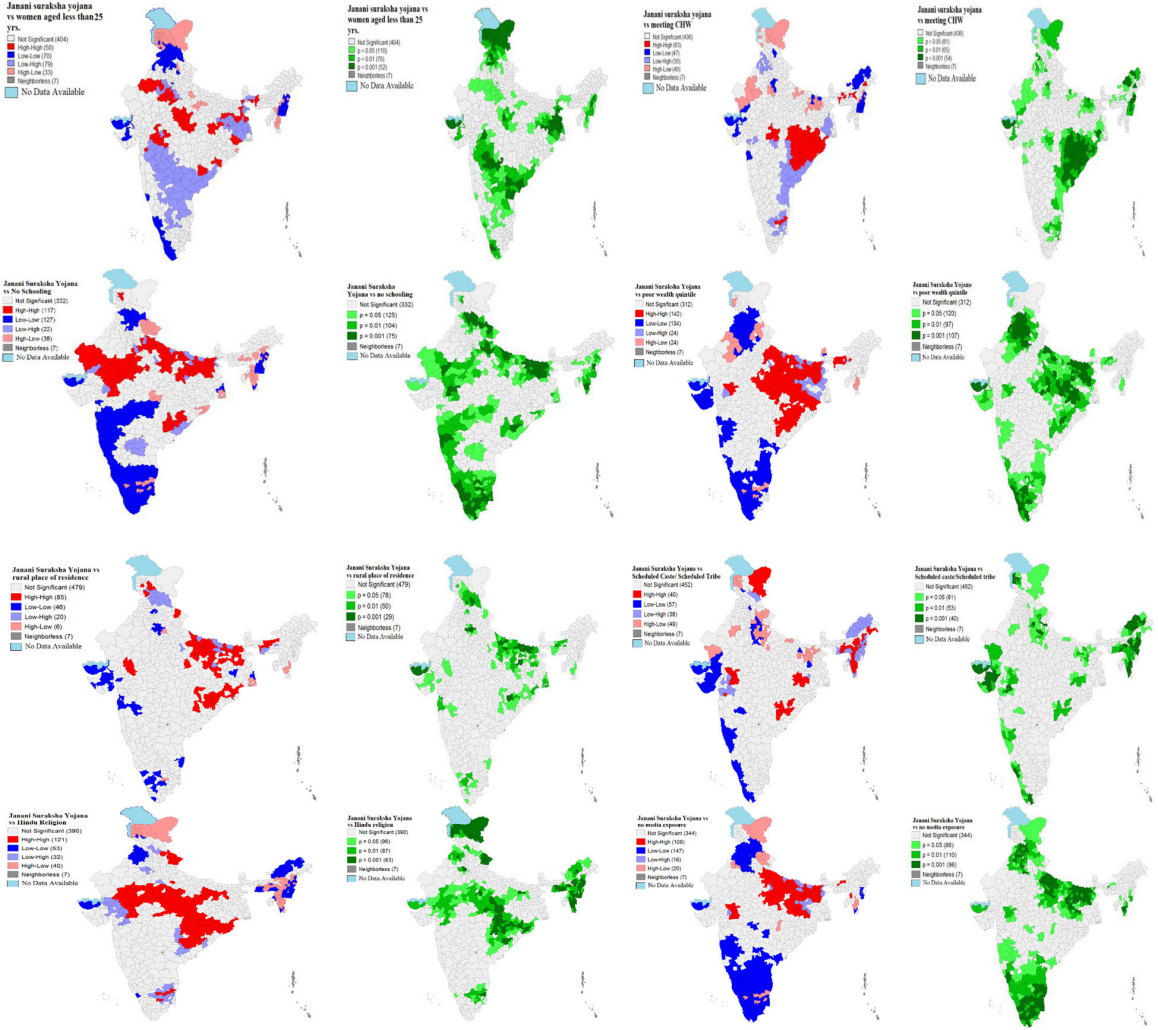
Bivariate Local Indicator of Spatial Association (BiLISA) (cluster and significance) maps for dependent vs outcome variables for districts of India, 2015–16. *CHW: Community health worker; Yrs.: Years*

Figure 3 displays the bivariate LISA cluster map which indicated the high-high clustering for JSY and women aged less than 25 years were observed in 50 districts which were from Rajasthan, Madhya Pradesh, Odisha, West Bengal, Jharkhand and Chhattisgarh. The hotspots (high-high) clustering for JSY and women who met CHW were observed in 63 districts which were from Madhya Pradesh, Odisha, Andhra Pradesh, West Bengal and Tamil Nadu.About117 of 640 districts had the highest JSY service utilization and no schooling among women. These districts mostly were from Rajasthan, Madhya Pradesh, and some parts of Uttar Pradesh, Bihar, and Odisha. However, cold spots (127 districts) of JSY utilization and no education were found in the southern part of India. Bivariate LISA map suggested that around 142 districts constitute the hot spots of high JSY utilization and high poverty. Majority of these districts were from Uttar Pradesh, Bihar, Jharkhand, Chhattisgarh, Odisha, West Bengal, and Assam. Only 85 districts constitute the hot spots of high JSY coverage and rural areas. These districts were from Uttar Pradesh, Bihar, and few districts from Chhattisgarh, Odisha, and Assam. About 20% of districts (121 districts) of India were observed as hot spots (high JSY utilization and high Hindu religion population) while 53 districts were found as cold spots (low JSY coverage and low Hindu population). Mostly hot spots districts were from Madhya Pradesh, Chhattisgarh, Odisha, and some part of Uttar Pradesh and Uttarakhand. Similarly, around 109 districts were identified as hot spots (high JSY coverage and high no mass media exposure) and 147 districts as cold spots (low JSY coverage and low no mass media coverage). These hot spots district from Uttar Pradesh, Bihar, Jharkhand, and few districts from Madhya Pradesh whereas cold spots were found in the southern part of India and the states of Punjab Chandigarh, Haryana, and Himachal Pradesh.

## Discussion

The study found that 36.4% of women in India got benefited from Janani Suraksha Yojana (JSY). The value of Moran’s I was 0.71 which depicts a high spatial auto-correlation i.e., the high dependence of the JSY variable over districts of India. Additionally, more than 50% of the women were utilizing JSY services in the districts of Odisha, Chhattisgarh, Madhya Pradesh, Uttar Pradesh, Uttarakhand, Assam, and few districts of Rajasthan, Bihar, Jharkhand, and Meghalaya. Lastly, it was revealed that women aged 25 years or less, women who met with CHW, women from poor wealth quintile and women with no schooling status were positively associated with JSY coverage among districts of India. Further, our bivariate findings also support the logistic regression analysis in the association with utilizing JSY services with the background factors. The confounder variables included in the logistic regression analysis were found significant.

Though this spatial analysis of the JSY coverage is an attempt to find the clustering of exposure to the programme at the district level in India, and the paper is acknowledged with several interesting findings. Firstly, there is a need to improve the policy coverage at the very household level so that, it can make thrive the concept of universal access and enhance the socio-spatial coverage too. Secondly, in receiving the JSY services by the beneficiary groups of people, there are multiple social determinants of health that affect accessing it and therefore need to be prioritized at the individual, household, and community levels. Thirdly, a high health inequity is seen across the spatial-regional distributional patterns of JSY service and it concentrated at one particular geographical region and it also extremely varied within and between regions.

In the present study, it was found that regional disparity was visible in the case of JSY coverage across districts of India. For instance, the concentration of JSY coverage among illiterate women was visible in states of Rajasthan, Madhya Pradesh, Uttar Pradesh, Bihar and Orissa which are considered as the empowered action group (EAG) [38] states. Moreover, the spatial auto-correlation of JSY and women from poor wealth status was concentrated in the entire central and eastern part of India which has lower socio-economic development in comparison to other parts of India [5, 20, 35, 52]. The regional disparity also remained the same in the coverage of the programme, although the financial incentives have led to the poor women in more service utilization under the NRHM policy [5, 21, 22, 29], but, there is evidence that shows the targeted groups are lacking in availing the services [11, 12] which need to be enhanced with the universalization of the programme to every individual.

There is evidence shown in previous studies that accessing maternal and child health-related information in the community as a whole and household particularly depend on several factors. Socially and economically marginalized communities receive fewer services as compared to their counterparts [13, 15, 38, 53]. At the same time, women belonging to these communities have faced discrimination in availing healthcare services [10, 28, 34]. In our analysis, it is shown that there are regions and districts which are socio-economically poor, and the proportionality with a high SCs and STs population are shown under-coverage of JSY services. For example, in the states like Jharkhand, Chhattisgarh, Bihar, and Madhya Pradesh, and Uttar Pradesh, the coverage of JSY across socio-economic groups are not up to that mark and it varied significantly across districts and regions too. The factors like governance, CHWs and associated determinants often played a role in the implementation of the programme and the community health workers that are key to make it a success. Previous studies have also supported our findings regarding the under-coverage of JSY scheme in some of the districts is due to unavailability of CHWs, lack of governance, and the interaction between stakeholders and CHWs [27, 39–41, 46, 54, 55]. Further, the major finding of this study is that the regions which were already facing high inequity in health service coverage yet again spotted with socio-spatial inequality in the JSY coverage. Overall, the high concentration region of JSY coverage is shown in the central region (states included as Madhya Pradesh, Uttar Pradesh, and Chhattisgarh), the eastern region (Bihar, Jharkhand, and Orissa) and the northern region (Uttarakhand and Rajasthan and Jammu & Kashmir). These regions or states are having huge health disparities that can be also seen in the distribution of JSY services [6, 36, 38, 56]. Contrary to that, the southern and western part of India is partially covering the programme, although there are some patches in Tamil Nadu (South Indian state) showed the coverage of the JSY. However, southern states are falling back with the programme coverage. Even though in the southern states, the private institutional deliveries have increased irrespective of the socio-economic conditions in the last decade [23], moreover, some households are eligible to get the JSY services, are still lacking access to the services. The regional inequality in the JSY coverage has also put women’s health at risk and therefore the regional planning and policy concern is highly required in India. The lower the coverage lesser the inequality, and higher the coverage the highest inequality is seen in the JSY utilization across India.

Regional inequality and high severity in social policies have made profound effects on MCH outcomes in many developing countries, including India [17–19, 27, 29, 52]. Historically, a lack of policy consistency and programme intervention on evidence-based maternal healthcare in India has made a lesser imperative in the development of mother and child health. Further, there has also been little intervention on socio-behavioral change in the community that paved to rural women to deliver their child at health institutions except for JSY that helps financially after the child born at public health institutions [4–6]. Although the programme was to support rural pregnant women to deliver their babies in public health institutions, however, the results show that there is still inequality in the distributional patterns of service utilization among the population who are eligible to adhere to it [57]. The findings of this study clearly show that the service utilization among the poor and disadvantaged groups of women is higher compared to their counterparts, even though the programme was for the targeted groups, however, the results showed that it is still lacking the full coverage of service utilization among them [35, 37, 38, 57, 58]. As the previous studies have also provided the evidence and supported the analysis in the context of service coverage where the women deprived of multiple grounds face inequity in the use of JSY service [38, 57]. Moreover, previous literature argued that after JSY in 2005–07, the benefit was more weighted towards rural, illiterate and women from lower socio-economic strata [34, 41, 55]. Additionally, it was too argued that the concentration of JSY coverage was high among women from lower socio-economic strata because of cash incentive system of JSY [5, 11, 21]. The use of public institutional delivery has increased many folds among the poor socio-economic women after the launch of the National Rural Health Mission (NRHM) in 2005 and it turned up as a pro-poor programme [9, 12, 20, 27]. However, the gap remained the same in accessing the JSY service (Under the NRHM) by marginalized and disadvantaged women which are shown in this study as well. The findings are also consistent with previous evidence that the probability of service utilization is more among those who are not deprived of multiple socio-economic and political grounds [7, 17, 34, 54].

Although the study had some limitations too, firstly, recent advancements and implementations under the JSY could not be analyzed as the data source used for 2015–16. Secondly, socio-economic inequality within districts was not assessed which would be interesting to evaluate in further analysis. Thirdly, the reasons why women cannot assess JSY benefits were not covered in the study which can be further investigated through qualitative research. Lastly, more magnification of the districts was not possible due to sample size issues at primary sampling unit’s (PSU’s) that would be of utmost importance for block level policy implementation.

However apart from the above limitations, the analysis provided a broad perspective regarding inequality in JSY coverage across districts of India which can be very important for policymakers to evaluate the scheme at district level.

## Conclusion

The overall utilization of JSY services in India after the launch of the programme in 2005 is still 36% only. The coverage of JSY highly varied across different regions, districts, and even socioeconomic groups. It was reflected that the high coverage of JSY was concentrated in EAG states and among poor and illiterate women. Yet, rural and remote areas with geographical barriers across Indian states and districts are shown under-coverage. There is a need to mobilize the resources and implement JSY in every corner of the districts of the country so that every woman should get benefit from JSY and to reduce inequality across districts. Because it has huge implications for mothers and child survival. No doubt, the JSY scheme in India has led to an increase in MCH services among pregnant women but due to unequal utilization of JSY service coverage across the groups made unequal distribution in the districts and states. Though the JSY programme has been less known among families, for example, those who had have no mass media exposure, no education, lower-caste groups, and poorer households. However, the JSY coverage is fair enough comparatively and it needs more attention from the government in response to implementation and governance. Also, to counter social determinants of women’s health a need-based policy intervention like this is required to enhance the MCH coverage among the poor and marginalized women. To make an effective such programme like JSY, the CHWs (community health workers) need to be trained and engaged for diffusing the information at the individual and household levels.

## Data Availability

The study utilizes a secondary source of data that is freely available in the public domain through http://iipsindia.org.

## Abbreviations

JSY: Janani Suraksha Yojana
MCH: Mother and Child Health
NRHM: National Rural Health Mission
EAG: Empowered Action Group
CHW: Community Health Worker
BPL: Below Poverty Line
ASHA: Accredited Social Health Activist
NFHS: National Family Health Survey
MoHFW: Ministry of Health and Family Welfare
PPS: Probability Proportional to Size
HH: Household
CEBs: Census enumeration block
LISA: Local indicators of spatial association
OLS: Ordinary Least Square
SLM: Spatial Lag Model
SEM: Spatial Error Model
AIC: Akaike information criterion
